# Preparing Advanced Clinicians and Practitioners: A Model for Mentorship in Occupational Therapy Practice

**DOI:** 10.1155/2021/3394478

**Published:** 2021-12-21

**Authors:** Sarah A. Schoen, Bryan M. Gee, Mim Ochsenbein

**Affiliations:** ^1^STAR Institute, Centennial, Colorado 80112, USA; ^2^Post Professional Doctor of Occupational Therapy Program, Rocky Mountain University of Health Professions, Provo, Utah 84606, USA

## Abstract

Mentoring is essential at all stages of a professional career. However, little has been written about the effectiveness of programs for practicing clinicians. This study was designed to address the need for evidence about the effectiveness of formal mentorship programs by describing the impact of the STAR mentorship program on a group of clinicians specializing in sensory integration and processing challenges. This study utilized an exploratory, retrospective, survey research design. Course evaluations were examined from 240 subjects following participation in a one-week, small group mentorship training program. Qualitative methods were adapted for use in this study. Sixteen codes, with operational definitions, were developed to analyze the surveys. Ninety-six percent indicated that the program met or exceeded their expectations; only 12.5% had a negative comment. *Impact on psychosocial function* was reflected by 22% of the participants. Comments related to *impact on career function* were indicated by 45% of the participants. Ninety-four percent provided *positive* comments about the program *structure*, and 74% responded with *positive* comments regarding *content* of the program. Positive outcomes were associated with this one mentorship program, suggesting a need for more in-person, structured mentored learning experiences. Mentorship is recommended as a method to address the growing need within the profession to support career development, build knowledge, skill and attitudes, and aspirations/commitment as well as enhance professionalism/professional development.

## 1. Introduction

Mentoring is an essential component of professional growth and development both in the early stages and later stages of a professional career. The importance of mentorship is recognized within health care professionals in general [1] as well as within an increasing body of literature recognizing its value/import within the field of occupational therapy specifically [2]. Beyond the classroom, mentoring serves to help students and new graduates establish their professional identity [2] as well as attitudes related to professionalism and professional commitment [3]. In the middle stages of a career, mentoring serves to increase job satisfaction, improve professional confidence, support achievement of professional goals and skills, and increase opportunities for networking within the profession [4]. In later years, mentoring solidifies knowledge/creates collaborations between mentor and mentee and improves competency in significant areas of practice [1, 5].

Occupational therapy (OT) entry-level education is filled with opportunities for experiential learning. However, once one completes an academic program, fewer opportunities exist for participation in mentored experiences. Additionally, clinicians are increasingly more isolated because of service provision settings (e.g., rural areas or private practice). Research suggests that rural health care providers have greater difficulty accessing educational opportunities thus impacting knowledge and skill development in these professions [6].

Although mentoring within allied health professions is not a new concept, it has resurged over the past 10-15 years [1, 4]. Little however has been written about the nature of mentorship for practicing clinicians, programs designed to promote advancement, and the effectiveness of these programs. A recent review article identified methodological weaknesses in the existing literature such as a lack of uniform definitions or rigorous research designs and a scarcity of standardized measures to evaluate effectiveness of mentoring programs [2].

Starting in 2007, the SPD Foundation (now known as STAR Institute) identified a need for mentoring opportunities for occupational therapists working in pediatric settings. These unique one-week mentorship training programs were initially designed for experienced clinicians specializing in sensory integration and relationship-based practices. Both sensory integration and relationship-based practices are specialized interventions for children with sensory processing challenges that impact a range of developmental disorders such as ADHD, autism, and learning disabilities. This integrated treatment approach is based on theories of sensory integration originating from the work of Dr. A. Jean Ayres [7] as well as theories related to relationship focused intervention based on the work of Greenspan and Weider [8]. Level 1 mentorship utilizes a small group format and is the prerequisite for other more advanced trainings. Programs involve a combination of classroom instruction, discussion, and reflection based on on-site clinical learning experiences.

### 1.1. Definition of Mentorship

Mentorship is defined as a working alliance between individuals in which the more experienced people support the growth and development of those with less experience [1, 9]. Mentorship involves several aspects including teaching, guiding, and support. Mentors advise, tutor, counsel, and coach but play a crucial role in contributing to the evolution of another's career and professional identity [10]. The intent of the relationship is to develop and enhance knowledge and skills as well as share insights that have been learned throughout the years [11]. Mentoring describes how a mentor facilitates personal and professional growth while at the same time cultivating wisdom [12]. Thus, a hallmark distinction between mentor and the person being mentored is “degree of development,” such as life experience, age, or work experience.

### 1.2. Mentorship Is Different from Supervision

Mentorship is based on a mutual desire for career development for the purpose of updating one's professional skills. It capitalizes on a nonreporting relationship, a relationship within which there is no accountability or performance evaluation. Conversely, the purpose of supervision is to ensure that an employee's service and performance are congruent with their job description. Supervision helps to shape one's behavior so that organizational and professional goals are met, regarding job identity, competence, skills, and ethics [13]. Often supervision is provided for new therapists or employees and in some settings may even stop after a certain amount of time.

### 1.3. The Functions of Mentorship

Mentorship is defined as a relationship that is close, personal, nurturing, and dynamic [14]. It is nonauthoritarian in nature offering guidance, support, and encouragement towards professional development and improvement of the mentee [15]. It has been described as having two primary functions [13, 16]. Mentorship has a *career function* by exposing the individual to new professional arenas, providing resources and new knowledge. It also has a *psychosocial function* by cultivating competencies, achievement, and motivation of the mentee through the mechanism of a supportive and caring relationship as well as creating opportunities for networking among colleagues [16, 17].

The literature points to mentoring as being essential to professional growth and development. It is a mechanism for establishing professional identify as well as a vehicle toward increasing job satisfaction and professional confidence. Importantly, as job satisfaction increases, so too does one's commitment to their career [14, 18, 19].

The current state of knowledge in occupational therapy suggests that experiential opportunities are more abundant for entry-level clinicians, and that practicing therapists may be increasingly more isolated. Small group mentorship experiences are not often discussed in the literature. Programs that are described tend to fall into one of three areas: mentorship for occupational therapy students during fieldwork [20, 21], mentorship for occupational therapy faculty [22, 23], and mentorship of new faculty within academic programs [2].

However, less is written about mentorship for practicing occupational therapy clinicians. In fact, in a scoping review of mentorship programs for occupational therapists, only seven out of twenty articles reviewed described programs directed toward clinicians. Within those that were reviewed, only seven were clinician-focused programs, with even fewer focused on pediatric clinicians. Several utilized a peer comentoring structure rather than the more traditional hierarchical relationship where the mentor is more senior and more experienced in a particular area relevant to the career development of the mentee [2].

There is recognition that those working in specialty practice areas such as sensory integration require further learning and clinical mentoring to become experts [3, 23]. However, the benefits of mentoring have not been fully studied in terms of impact on clinical skills as well as other potential benefits (e.g., professional competency, professional confidence, and professional attitudes). One study reflecting the perspective of mentors in sensory integration confirms the long-held belief of the importance of mentoring for enhancing attitudes of professionalism and professional commitment [3].

This current study was designed to address the need for evidence about the effectiveness of formal mentorship programs, mentorship groups, and mentorship in occupational therapy.

### 1.4. Description of the STAR Institute Mentorship Program

The STAR Institute established its mentorship program in 2007 under the name of the SPD Foundation. These small group experiences allowed for classroom instruction discussion and reflection. Each program lasted for one week and engaged between 12 and 14 learners/mentees in the program. The structure was both didactic and experiential with exposure to such topics as assessment of sensory integration and sensory processing, goal writing, clinical reasoning used in treatment planning, documentation, and caregiver education. When not in the classroom, learners were able to observe live treatment sessions through a one-way mirror with microphones to capture audio exchanges among therapist, child, and parent. During and following the observation sessions, mentors and mentees engaged in clinical reasoning discussions based on the child's symptom presentation relative to sensory-related behaviors impacting participation in daily life activities.

### 1.5. Purpose of the Study

Thus, the purpose of this study was to describe the impact of the STAR mentorship program on a group of pediatric occupational therapists. Specifically, the objective was to explore the perceptions of clinicians specializing in sensory integration and processing. Feedback from the learners was designed to tap both strengths and weaknesses of the mentorship program.

## 2. Materials and Methods

This was an exploratory, retrospective, survey research design, which examined course evaluations following participation in a one-week, small group mentorship training program. The protocol was reviewed by the Rocky Mountain University of Health Professions, Provo, Utah Institutional Review Board who deemed it to be exempt.

### 2.1. Description of Participants

Participants were occupational therapy clinicians who completed the level 1 mentorship program at STAR Institute (previously known as SPD Foundation). All reported working with children or adults with sensory processing and integration challenges. Data is from 240 clinicians who attended the training from 2007 to 2014. This timeframe was selected for consistency of instructors, structure, and content. Surveys were completed anonymously so limited demographic data were collected; name, age, gender, and years in practice were unavailable. Additionally, a calculated response rate was not tracked as a part of this study.

### 2.2. Data Collection

At the end of each mentorship week, participants completed an overall course evaluation used for quality assurance/management at a program implementation level, which served as the research questionnaire survey for this study. Data were collected anonymously from participants. The survey contained seven open-ended questions that asked for participants' opinions of or perspectives on their learning experiences, strengths, and weaknesses of the program and suggested changes. The questions are listed below:

1. Did mentorship meet your expectations?
2. Did you feel the mentorship was a good value for what you received?
3. What other topics or subject matter would you have liked to see covered this week?
4. What did you like best about the mentorship program?
5. What did you like least about the mentorship program?
6. What would you suggest we do differently in the future?
7. Other comments about this program

### 2.3. Data Analysis

Qualitative methods were adapted for use in this study. The two approaches used for coding were inductive and deductive methods. The first was an inductive process that was data driven. This approach involved looking at the feedback in the text without a predetermined conceptualization [17], e.g., letting the text speak for itself.

Fifteen participant responses were first reviewed to get an overall sense of the data. From this review, a list of codes emerged based on this sample of the responses. The process of inductive coding followed recommended procedures [17]. The dataset was divided into smaller samples. After an initial and thorough reading of a sample of the data, patterns or themes were written down. Two research assistants searched for patterns and consistency across respondents. Codes were reviewed with the first author to ensure agreement was reached amongst the team. Codes were created and applied to that sample. Each code was assigned an operational definition, with an effort made to identify passages of the text that shared the same code to provide examples in the operational definition for each code. Codes were then applied to the rest of the data. If indicated, codes were modified, clarified, or consolidated based on the rest of the responses. There were a total of 16 codes that were agreed upon and subsequently used by two research assistants to analyze the remaining responses, while in a rare instance, when required, some of the responses were recoded.

Ten percent of the sample (e.g., *n* = 24) were scored for interrater reliability. Interrater reliability was calculated using the following formula: agreement/(agreement + disagreement) × 100. Scores ranged from 75% to 97% across this sample. Frequencies of code endorsement by participant (e.g., related to program evaluation) were then calculated. Descriptive statistics were used to summarize and tabulate the data.

We then applied a deductive process that was concept driven [17]. These concepts were taken from the literature. Codes were reduced and simplified into broader categories reflecting: (1) the association with the two types of mentoring proposed by Kamm [16] who differentiated *career function* from *psychosocial function*, (2) whether the comment was *positive* or *negative*, and (3) if *positive* comments were related to the *structure* of the program versus those related to the *content* of the program. Frequencies for these category analyses were also calculated.

## 3. Results and Discussion

Results are reported in Table 1. Responses were generally positive. Participants identified strengths of the program as well as suggestions for change. Positive feedback was obtained from 95.83% of participants, indicating that the program met or exceeded their expectations; only 12.5% had a negative comment.

Six broader categories were produced from the sixteen codes in Table 1. These categories were designed to reflect the two primary functions of mentorship, psychosocial and career function. Codes that comprised the psychosocial function category were as follows: supported personal reflection, opportunities for networking, and allowed expression of ideas. Codes that comprised the career function category were as follows: provided resources, linked theory to practice, benefited from clinical observation, and benefited from clinical reasoning. Additionally, comments were grouped to reflect positive comments about the structure and positive comments related to the content of the program (see Table 2).

### 3.1. Psychosocial and Career Function

When examined with respect to *career* and *psychosocial* function, the following was found: *impact on psychosocial function* was reflected by 22.08% of the participants. Specific influences were reported on professional identity as well as notably that the mentorship program instilled confidence, positively affected their professional outlook, and that they appreciated networking with colleagues. Comments related to *impact on career function* indicated that 45.41% indicated that the training had a specific influence on clinical skills such as clinical observations, clinical reasoning, translating theory to practice, and access to resources.

### 3.2. Structure and Content

*Positive* comments about the program *structure* were provided by 97.73% of participants reflecting the organization of materials, topics, and pace of interaction with participants and faculty. *Positive* comments regarding *content* of the program were expressed by 73.75% of the participants while *negative* comments were expressed by 12.5%., Those with negative comments felt that the program did not meet or partially met expectations, highlighting that the course was too expensive. *Negative* comments were related to both the *structure* of the program and program *content*. Specifically, suggestions were to restructure the program by changing the pace of lectures, grouping participants based on level of experience and more time for breaks. Suggestions also included revising contents by addressing specific content areas by either spending more time on some topics and less time on other topics. There were no negative statements related to professional identify or impact on clinical skills, although participants indicated wanting to schedule one to one meeting with mentors and to wanting more time to address issues related to interdisciplinary treatment.

*A sample of comments included*: “mentors were great resources”; “the program facilitated clinical reasoning” and “supported integration of theory into practice”; “the environment welcomed discussion and expression of opinions,” and “it was a great opportunity for networking with other clinicians.” Areas for improvement included the addition of topics related to practice setting (e.g., school-based clinicians wanted more information about application of knowledge to school settings). Those with recommendations for change suggested more mentor-guided independent learning opportunities and smaller group analyses of videotaped treatment sessions.

## 4. Discussion

Overall, clinicians regarded this mentorship program as a positive and valuable experience. Mentees enjoyed the format of learning experiences and the unique opportunity to network with other practicing clinicians. Responses were similar to those expressed by occupational therapy students in a study at the University of Western Ontario, Canada [2], suggesting that small group mentoring may be beneficial at all stages of one's professional career. In general, research on the effectiveness of mentorship supports the improvements reported in this study, which included gains in attitude, motivation, and organizational commitment, but equal in job performance, job productivity, and job success [2]. One program only showed changes related to intervention reflected by increased clinical skills, information provision, and treatment self-confidence [24].

The program described in this study fulfilled many of the projected responsibilities of a successful mentor articulated by Roberson and Savlo [13] by fostering reflection, providing instruction and reflection, supporting critical thinking and clinical reasoning, facilitating problem solving and decision-making, and encouraging application of learning. Importantly, this program was also found to align with all of the key components of American Occupational Therapy Association's (AOTA) standards for continuing competence by supporting growth and development in knowledge, critical reasoning, interpersonal abilities, performance skills, and ethical reasoning [25, 26].

Implications can be gleaned from this study for both entry-level academic education and postgraduation professional development. In an age in which distance education via online synchronous or asynchronous learning is growing and technology is increasingly a part of educational experiences, there is a recognized need for in-person, structured mentored learning experiences that utilize a hybrid approach [27]. Participants in this study valued the face-to-face contact with mentors and opportunity to observe/interact with peers/colleagues as well as clients in the clinic.

Based upon the results of this study, small group mentoring seems to be beneficial at all stages of one's career. The mentorship provided through this program seemed to foster the development of professional expertise as well as instilling a sense of commitment among attendees to the profession. All participants described some benefit consistent with mentoring. Results suggest more mentorship programs like the one described in this article would be beneficial to the occupational therapy profession.

As recognized in the existing literature [13], mentorship programs are shown to be an effective method for addressing the growing need within the profession to support career development, build knowledge, skill and attitudes, and aspirations as well as enhance professionalism/professional development. The mentoring provided in this program seemed to be an important mechanism for developing a commitment to the profession, specifically with respect to pediatric intervention for children with sensory processing and integration challenges. This is consistent with other research over the past five years describing equally innovative programs of mentorship [5, 16]. Mentoring programs in the occupational therapy profession have demonstrated outcomes related clinical skills, research skills, interprofessional teamwork, professional presentations, and papers as well as communities of practice and a sense of belonging [2]. Results of the current study suggest that the mentorship program described here fulfills many of these same outcomes.

Clearly, more research is needed into the efficacy of mentorship programs relative to their effectiveness in supporting the development of occupational therapy practitioners across clinical populations and settings. Current research advocates for studies to address methodological gaps, with a need for occupational therapy practitioners and researchers to continue researching mentoring experiences using more rigorous designs and standardized measures of program effectiveness [2].

### 4.1. Study Limitations

This study had several limitations. No demographic data was collected on the participants. Evaluations were completed anonymously so there was also a lack of information on experience levels, years in field of sensory integration and sensory processing, or years in the profession. Thus, there is no data about the characteristics of each mentorship small group cohort: specifically, it is unclear as to which groups had a more homogenous sample of participants.

Additionally, all data were collected retrospectively. There was no scheduled opportunity to follow up with respondents to determine how the experience in mentorship has altered their professional life or what components of the program they were able to implement in their own practice. The survey tool that was used for data collection in this study was not developed for the purpose of research so questions may not have tapped all areas of interest to program development or the impact of the program on career or psychosocial functions specifically. Future prospective studies need to be designed to explore intended impact of the program and intended applications.

Lastly, there were no questions related to the characteristics of the mentors that may have supported learning or differences across mentors responsible for teaching the content. A future topic of interest is to reflect on the benefits of being a mentor within such structured programs.

## 5. Conclusion

As a result of this study there is a recognized need for small group learning with recognition that observations (either onsite or virtual) in the clinic setting are beneficial. There is a clear benefit of mentoring opportunities for both novice and experienced health care professionals. Ultimately, mentorship strengthens the integrity and viability of specialty areas as well as to the profession as a whole.

## Figures and Tables

**Table 1:** Percentage endorsement based on codes.

Codes	Mentee response (*N* = 240)	
	*n*	%
Positive feedback	230	95.83
Provided resources	51	21.25
Linked theory to practice	66	27.50
Supported personal reflection	53	22.08
Opportunities for networking	10	4.17
Allowed expression of ideas	48	20.00
Benefitted from clinical observation	107	44.58
Benefitted from clinical reasoning	35	14.58
Valuable presentations	225	93.75
Negative feedback	30	12.50
Positive impression suggested time inc/dec	140	58.33
Need to gear content to specific practice setting	6	2.50
Program structure	163	67.92
Recommend program structure change	11	4.58
Miscellaneous logistical feedback	50	20.83
Negative impression (re: time spent on topics)	9	3.75

**Table 2:** Percentage endorsement based on category.

Category	Mentee response (*N* = 240)	
	*n*	%
General positive statements	230	95.83
Impact on career function	109	45.41
Impact on psychosocial function	53	22.08
Positive comments related to structure	225	93.73
Positive comments related to content	177	73.75
General negative statements	48	12.50

## Data Availability

The data used to support the findings of this study are available from the corresponding author upon reasonable request.
